# It’s Your Game-Tech: Toward Sexual Health in the Digital Age

**DOI:** 10.4236/ce.2014.515161

**Published:** 2014-08

**Authors:** Ross Shegog, Melissa F. Peskin, Christine Markham, Melanie Thiel, Efrat Karny, Robert C. Addy, Kimberly A. Johnson, Susan Tortolero

**Affiliations:** Center for Health Promotion & Prevention Research, The University of Texas School of Public Health, Houston, TX, USA

**Keywords:** Adolescents, Web-Based Health Education, Computer-Based Health Education, Health Communications, School-Based Health, Sexual Health

## Abstract

Adolescent sexually transmitted infection (STI) and birth rates indicate a need for effective middle school HIV/STI, and pregnancy prevention curricula to delay, or mitigate consequences of, early sexual activity. Individual and organizational barriers to adoption, implementation, and maintenance, however, can hamper dissemination of evidence-based sexual health curricula, adversely impacting fidelity and reach. Internet-based approaches may help mitigate these barriers. This paper describes the development and feasibility testing of *It’s Your Game* (*IYG*)-Tech, a stand-alone 13-lesson Internet-based sexual health life-skills curriculum adapted from an existing effective sexual health curriculum—It’s Your Game… Keep it Real (*IYG*). *IYG*-Tech development adaptation steps were to: 1) Select a suitable effective program and gather the original program materials; 2) Develop “proof of concept” lessons and test usability and impact; 3) Develop the program design document describing the core content, scope, and methods and strategies; and 4) produce the new program. Lab- and school-based tests with middle school students demonstrated high ratings on usability parameters and immediate impact on selected psychosocial factors related to sexual behavior—perceptions of friends’ beliefs, reasons for not having sex, condom use self-efficacy, abstinence intentions, negotiating with others to protect personal rules, and improved knowledge about what constitutes healthy relationships (all *p* < .05). Youth rated *IYG*-Tech is favorably compared to other learning channels (>76.2% agreement) and rated the lessons as helpful in making healthy choices, selecting personal rules, detecting challenges to those rules, and protecting personal rules through negotiation and refusal skills (89.5% – 100%). Further efficacy testing is indicated for *IYG*-Tech as a potential strategy to deliver effective HIV/STI, and pregnancy prevention to middle school youth.

## 1. Introduction

Adolescent births and sexually transmitted infections (STIs) represent serious public health concerns in the United States. Despite recent declines, the 2012 birth rate of women 15 – 19 years of age is 29.4 births per 1000 in the United States (Martin et al., 2013). The prevalence rate for at least one STI is 24.1% among adolescent females (aged 14 – 19 years) (Forhan et al., 2009) and 37.7% among those who are sexually experienced. These statistics substantiate the need for effective HIV, STI, and pregnancy prevention interventions for adolescents, beginning at the middle school level. There are at least five sexual health programs developed for use in the middle school setting with demonstrated effectiveness at delaying sexual behavior among middle school students (USDHHS, 2013). Despite this, many students are not receiving timely sexual health education (Martinez et al., 2010; Darroch et al., 2000) and many middle and high schools are not implementing evidence-based programs (Kirby, 2002) or requiring pregnancy and HIV prevention as health topics (Kann et al., 2007). Irrespective of their effectiveness, dissemination of such programs may be hampered due to barriers at the level of the program, individual, or organization (Kirby, 2007; Rolleri, et al., 2008; Noar et al., 2009).

Program barriers may include the difficulty to locate, obtain, and implement programs (Johnson, unpublished; Burlingame, 2011) or lack of sufficient training from program developers (Schaalma, 2004: 32; CDC, 2011: 57). Individual instructor barriers may include lack of awareness or confusion regarding education policies (Levesque, 2000; Peskin 2011), length of service in teaching related to resignation to attempt implementation (Markham, 2002: 40), lack of skills and self-efficacy for teaching and adapting programs with fidelity or negative attitudes towards “science-based” approaches (Rolleri et al., 2008; Buston, 2002). Organizational barriers include concerns of school officials about negative community reactions and perceived controversy of sex education (Landry et al., 2003), perceived lack of support from internal (e.g. administrator) or external (e.g. parent) stakeholders to teach sexual health (Rolleri et al., 2008; Peskin, 2014), pressure to devote class time to academic priorities rather than health topics, and lack of funds for purchasing, training, and implementing programs (Rolleri et al., 2008). A curriculum accessed through the Internet that includes individual interactive student activities can potentially address these program, individual, and organizational barriers by providing a channel to confidentially address sensitive material in a way that optimizes program fidelity (provided all lessons are received) in an easily accessible way that requires minimal teacher training and cost.

Policy and infrastructure initiatives are improving Internet connectivity to schools. Despite variability in reliable connectivity and bandwidth the Internet is a timely dissemination channel for sexual health interventions that promise to be educationally effective, cost-effective, and of high fidelity (National Center for Education Statistics, 2008). Evaluations of a computer-based sexual health intervention for high school students have demonstrated significant increases in knowledge regarding pregnancy, condom self-efficacy, attitudes toward abstinence, and perceived HIV susceptibility (Roberto et al., 2005), as well as decreases in frequency of sexual intercourse and number of sexual partners (Lightfoot et al., 2007). Recent literature reviews have indicated the efficacy of computer-based applications in impacting health behavior change in youth (Papastergiou, 2009; Primack et al., 2012; Hieftje et al., 2013). Methods describing the adaptation of effective group-based programs into programs delivered primarily by computer have been described (Card et al., 2011a, 2011b). The push for dissemination of evidence-based programs and the concurrent up swell in the potential of eHealth applications for behavioral change suggests the utility of adapting existing conventional programs to electronic channels.

*IYG*-Tech, a 13-lesson Internet-based curriculum, was adapted from It’s Your Game… Keep it Real (IYG), an effective middle school sexual health curriculum that has been shown to delay sexual behavior in two randomized trials (Tortolero, 2010; Markham, 2012). Adaptation challenges included ensuring that original learning objectives were fully represented, that a new Internet-based functionality of the original computer-based lessons could be achieved, and that the newly developed “proof-of-concept” lessons and prototype curriculum were acceptable to end users, feasible for delivery in the middle school setting, and impactful of determinants of sexual behavior. The purpose of this article is to describe a pragmatic and empirically-based adaptive development of *IYG*-Tech and results of usability and feasibility testing to determine readiness for school-based dissemination for efficacy testing. The empirical process described has broader implications for those interested in adapting existing evidence-based sexual health programs into computer-based applications.

## 2. Developmental Process: Methods and Results

Systematic translation frameworks informed our adaptation and development process, providing guidance on the incorporation of theoretically and empirically-based evidence (Bartholomew, 2006; Card, 2011b; Wingood & DiClemente, 2008). The development of the Internet-based HIV, STI, and pregnancy prevention curriculum (*IYG*-Tech) comprised two phases: 1) Adaptation of an existing hybrid (classroom/computer) evidence-based program (It’s Your Game… Keep it Real) into a completely Internet-based curriculum, and 2) Evaluation of the new program in a feasibility pilot test. The Adaptation phase approximated the stepped sequence described by Card et al. (2011) and the development of behavioral change matrices in the Intervention Mapping approach described by Bartholomew et al. (2006). An active participatory research process was instituted in accordance with the adaptation frameworks where a teen-advisory board provided information and advice regarding the translation of existing lessons and the development of new activities in consecutive meetings throughout the development phases.

### 2.1. PHASE 1: Adaptation

**Figure 1:**
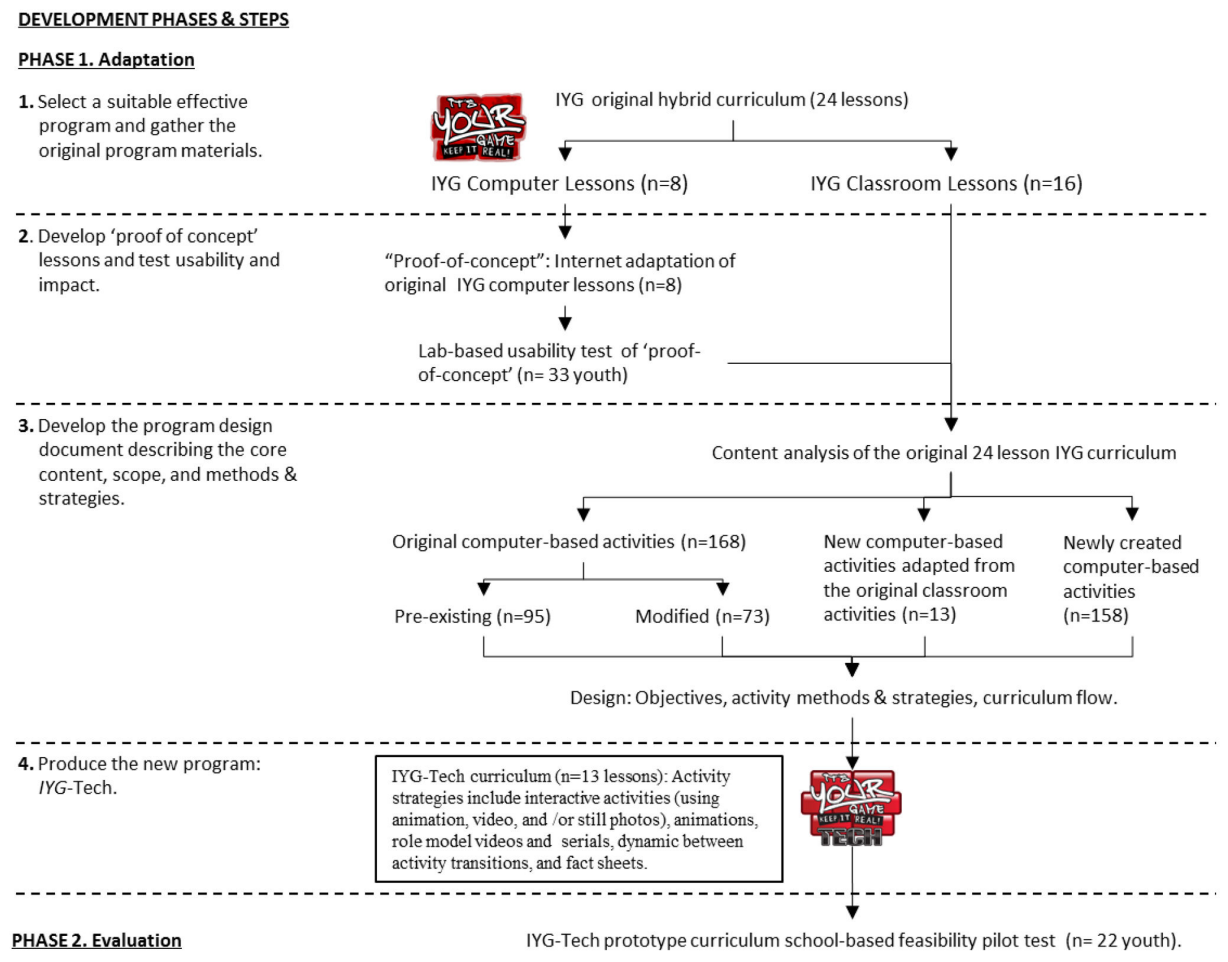
Conceptual framework of behavioral determinants and outcomes for IYG.

In the context of *IYG*-Tech Project the adaptation steps included to (Figure 1):

1. Select a suitable effective program and gather the original program materials;
2. Develop “proof of concept” lessons and testing usability and impact;
3. Develop the program design document describing the core content, scope, and methods and strategies; and
4. Produce the new program.

#### Step 1. Select a suitable effective program and gather the original program materials

**Figure 2:**
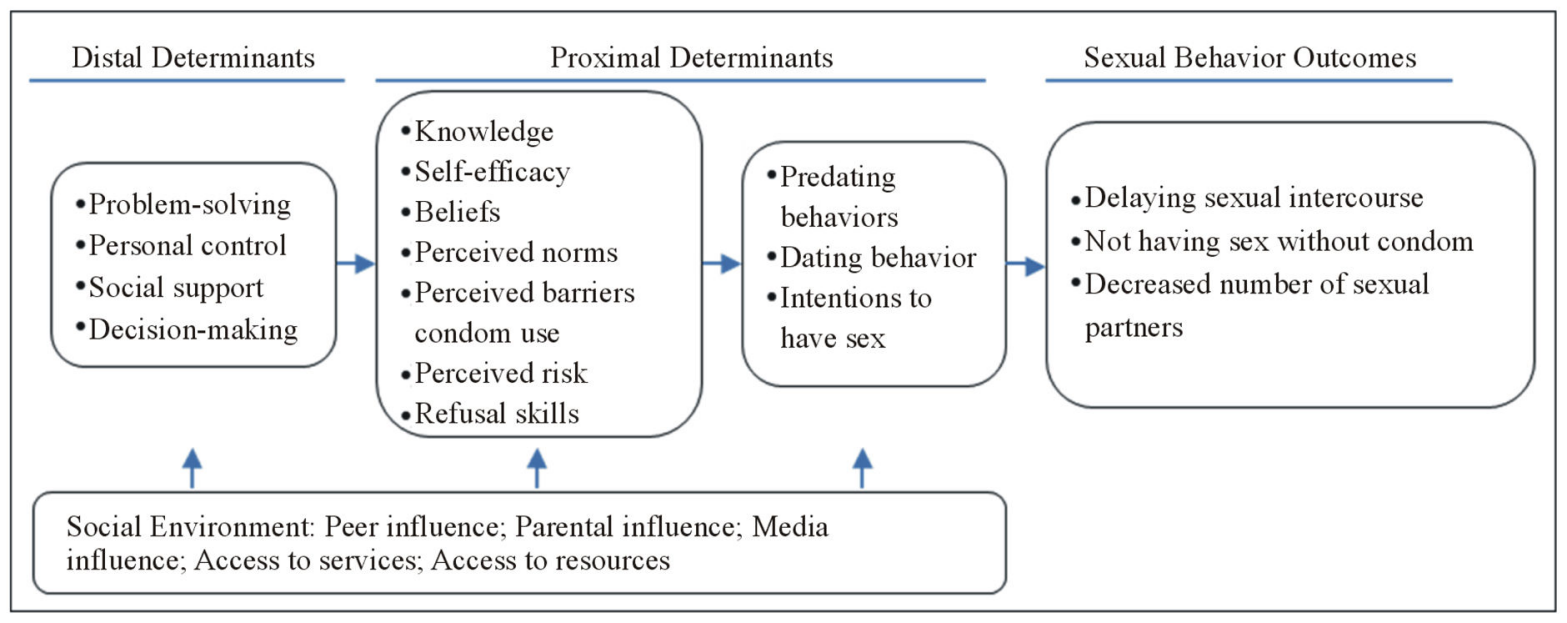
Conceptual framework of behavioral determinants and outcomes for IYG.

Adaptation is optimized through consultation with the original developers and assets of the original intervention (Card, 2011). The *IYG*-Tech curriculum was adapted from *It’s Your Game… Keep It Real* (*IYG*), an effective HIV, STI, and pregnancy prevention curriculum designed for middle school youth. IYG had demonstrated effectiveness in significantly delaying sexual initiation among sexually inexperienced students in two randomized controlled trials (Markham et al., 2012; Tortolero et al., 2010). Based on social cognitive theory (Bandura, 1985), social influence models (Komro et al., 2001; Perry, 1999; Story et al., 2002), and the theory of triadic influence (Flay & Petratis, 1994), *IYG* comprises 24 lessons—12 lessons each in 7th and 8th grade (Figure 2). For each grade level, *IYG* integrates 8 lessons of group-based classroom activities (e.g., role plays, small group discussion, and personalized journaling) and 4 lessons of computer-based, tailored multimedia activities (Shegog et al., 2007). A life-skills decision-making paradigm (*Select*, *Detect*, *Protect*) underlies all *IYG* activities, teaching students to *select* personal rules regarding risk behaviors, to *detect* signs or situations that might challenge these rules, and to use refusal skills to *protect* these rules. The IYG curriculum research and development team worked on the adaptation process for *IYG* Tech, following validated development protocols, informed by empirical findings from the target population, and existing computer lesson assets.

#### Step 2. Develop “proof of concept” lessons and test usability and impact

A proof-of-concept is the demonstration of a preliminary design and/or prototype used to test the feasibility of a program or product before it is more fully developed. While shown effective, the randomized controlled trials of IYG were unable to provide information on the relative effects of program components such as group process, individual computer lessons, and journaling activities. Prior to developing *IYG*-Tech, a usability study was conducted to determine the immediate psychosocial impact of the original 8 IYG computer-based lessons in order to understand the needed scope for a broader computer-based curriculum. These lessons were redesigned as a “proof-of-concept” for an Internet-based application to determine acceptability and feasibility of adjustments in look, feel, and functionality.

### 2.2. Translation of Existing Computer-Based Activities to an Internet Delivery Platform

The original computer-based lessons of the *IYG* curriculum had been delivered from the hard drives of dedicated laptop computers. The original *IYG* interface featured a 3-dimensional (3-D) virtual “mall” with first-person “shooter” navigation game dynamics. Increasing the dissemination potential of the program necessitated changes to the lesson interface, file compression, and image resolution. A “proof-of-concept” was developed to establish that original lesson content could be translated to minimize platform and bandwidth and to optimize Internet accessibility to schools. The look and feel of the original 3-D *IYG* mall interface and the active user control over navigation was replaced with a 2-dimensional (2-D) Flash-based interface and passive automatic user relocation within the *IYG* “mall” environment. Video components were compressed in size and embedded in Adobe Flash format to optimize accessibility, with some degradation of resolution, and interactive 3-D elements were replaced with 2-D Flash interactive elements.

### 2.3. Usability Testing

#### 

##### Study Design and Participants

A single-group, pre-test/post-test usability study of the 8 “proof-of-concept” Internet-adapted *IYG* lessons was conducted at two local community organizations and at a large university in southeast Texas, with a convenience sample of 7th- and 8th-grade students (*N* = 33), 12 – 14 years of age (*M*_age_ = 13.9 ± 1.14 years), primarily female (58%), and African American (70%), recruited through local public middle schools in a large, urban school district in southeast Texas and through the university. The sample size was consistent with usability testing protocols that do not require statistical significance to determine major usability problems (Nielsen, 1993). Participation was voluntary; written parental consent and child assent were obtained. The study was approved by The University of Texas Health Science Center at Houston institutional review board (HSC-SPH-07-0251).

##### Study Protocol

The youth accessed all 8 lessons in a single day (one of three consecutive Saturdays) in a simulated classroom setting using laptop computers with headphones. Youth completed pre-tests (demographic, psychosocial, and attitudinal surveys) and then completed each lesson individually. At the end of each lesson, each student completed usability questionnaires regarding that lesson. Students also completed usability assessments of the lessons as a whole. Upon completion of all lesson activities, students also completed psychosocial and attitudinal surveys (post-tests) that were administered via the same laptop computers. Sessions were observed by study personnel who documented any problems (technical or content-related) and provided assistance as required. Students received a $50 gift card for their participation. Breakfast, lunch, and between-lesson breaks were provided. Compensation was also provided to external staff whose presence was needed at community organization sites.

##### Measures and analysis

###### Usability

Usability parameters including likability, credibility, acceptability, understandability, ease of use, motivational appeal, and perceived impact were assessed using adapted Likert scale ratings and open-ended response formats (Shegog et al., 2007). *Likability* was based on how much the youth liked different lesson activities and program elements entire program, pictures/colors, sounds, buttons, cartoons, videos, transitions using 5-point Likert scale ratings, ranging from “dislike a lot” to “like a lot”. *Credibility* was based on the perceived correctness of the content presented using a 3-point Likert scale rating of “right,” “wrong”, and “don’t know” and whether the content could be trusted (“yes”, “no”, and “don’t know”). *Ease of use* was based on perceived difficulty of the entire program and directions within the program (very easy, kind of easy, kind of hard, very hard) and if youth needed help from the teacher or another adult to use the Internet-adapted *IYG* lessons (yes, no). *Understandability* was based on whether youth could understand the words used (yes, no). *Acceptability* was based on the pace of Internet-adapted *IYG* activities (“too fast”, “just right”, “too slow”). Parameters of *understandability* of words used, *motivational appeal* (if youth would use these lessons again and recommend it to others), *ease of use* (if youth needed help from the teacher or another adult to use the adapted lessons), and *perceived impact* (if youth thought these lessons would help them make healthy decisions about friends and sexual relationships) were rated using a 3-point Likert scale (“yes”, “no”, and “don’t know”).

###### Psychosocial variables

Psychosocial mediators of sexual behavior were assessed to conduct exploratory tests of the impact of *IYG*-Tech and to establish feasibility for data collection and analysis for any impending efficacy field trial. The study sample, while sufficient for usability testing, is insufficiently powered to detect impact. However, exploratory analysis of mediators (which are the proximal targets of change) immediately subsequent to *IYG*-Tech provides an indication of the need for further lesson content above those in the original computer lessons. Psychosocial measures included beliefs about abstinence until marriage, beliefs about waiting to have sex, perceived friends’ beliefs about waiting to have sex, perceived friends’ sexual behaviors, reasons for and against having sex, self-efficacy to refuse sex, condom knowledge, condom attitudes, perceived friends’ beliefs about condoms, self-efficacy to use condoms, and perceived likelihood of having sex. All these psychosocial measures have been validated among multi-ethnic, urban-dwelling public school student populations (Basen-Engquist et al., 1999; Borawski et al., 2005; Coyle et al., 2004; Markham et al., 2012; Tortolero et al., 2010).

###### Attitudes toward computers for learning

Attitudes to computers were assessed to explore the impact of the *IYG*-Tech learning experience on global acceptance of computers as an educational medium. This is a broader measure of the motivational appeal of *IYG*-Tech, influencing the potential of seeing CAI in a new light. These attitudes were assessed using a 10-item survey, with a 3-point response scale and reported Cronbach’s alpha of .81 (Askar et al., 1992).

Usability measures were administered via paper-pencil surveys upon completion of relevant lessons. Psychosocial and attitudinal measures were administered via an audio-computer-assisted self-interview on laptop computers to enhance privacy and confidentiality. Data were analyzed using descriptive and inferential statistics (paired *t*-tests and sign tests), according to distribution assumptions, using SAS software (version 9.2).

##### Usability testing results

The resultant Internet-based lessons were 35 minutes in length and retained the original educational strategies of interactive 2-D exercises, quizzes, animations, peer videos, and fact sheets.

###### Usability

The Internet-adapted *IYG* lessons were rated as enjoyable (94%), easy to use (88%), and understandable (88%), with most students requiring no assistance (76%) (Table 1). Most students rated the lessons as credible (91%), useful in helping them to make healthy choices (91%), and of appropriate duration (66%). The ease of program directions, movement, and navigation were all highly rated (>90%). Most students indicated they would recommend the lessons to their classmates (79%), and some indicated they would do these lessons again (45%).

###### Psychosocial Impact

Immediately after completing the Internet-adapted *IYG* lessons, students perceived their friends as having more positive beliefs about waiting to have sex, cited more reasons for not having sex, had greater self-efficacy to use condoms, and had greater intentions to abstain from sex until marriage (*t*(23 to 30) = 2.390 to 3.589, *p* < .05). Students also reported that learning from computers increased their confidence and was very enjoyable (*t*(22) = 2.121 to 2.912, *p* < .05), but they also reported that using computers did not make the time pass more quickly nor allow them to learn more quickly (*t*(22) = −2.237 to −2.206, *p* < .05; Table 2).

The significant change observed in psychosocial mediators demonstrated the potential of the Internet adapted lessons. While positive, delivery of the Internet-based lessons impacted fewer psychosocial mediators than observed in the original field trial of the IYG curriculum (Tortolero, 2010). Additional mediators positively impacted in the field trial at 8th grade follow-up included more positive beliefs about abstinence, greater confidence in refusing sex, greater general knowledge about HIV and STIs, their symptoms, and using condoms to prevent them, exposure to fewer risky situations, fewer intentions to have oral sex in the next year, and greater intentions to remain abstinent through high school when compared to the comparison group (Tortolero, 2010).

Results of usability testing suggested that the “proof-of-concept” Internet-based lessons could be developed with acceptable usability ratings and positive impact despite down-grade from a 3-D interface in favor of reduced bandwidth. Usability ratings were similar to those of the original IYG computer program for credibility (91% vs. 92.9% for IYG), perceived effect of the program in helping make healthy choices (91% vs. 93% for IYG), and ease of use (88% – 100% vs. 78% – 100% for IYG). The adapted lessons rated lower on *understandability* (88% vs. 100% for IYG) with more students requiring assistance (24% vs. 0% for IYG). Conversely, appropriateness of time on task rated lower than the original IYG computer lessons (66% vs. 85.7% – 100% for IYG). A positive “by-product” of the current usability testing was the impact on attitudes toward technology for education in the classroom, a desirable but unexpected result from youth who are already sophisticated technology end-users. Collectively, these findings suggested the potential for the adapted Internet-based lessons as a feasible channel for sexual health education but that the original 8 IYG computer lessons would require supplemental content development to at least approximate the immediate impact of the original 24 lesson curriculum.

#### Step 3. Develop the program design document describing the core content, scope, and methods and strategies

A design document outlines the specifications (content, function, and flow) of a software product or, in the case of *IYG*-Tech, the proposed Internet-based curriculum. The design document, developed for the original *IYG* curriculum, to describe the program’s functionality, look and feel, screen maps, and scripts, was reviewed and amended to provide the “blue print” for production.

*Content analysis* of the original IYG curriculum helped to determine the scope of *IYG*-Tech. In the original IYG curriculum, the core content, scope, and best practice characteristics had been delineated using an Intervention Mapping (IM) process (Tortolero, 2010). These were represented in a series of behavioral change matrices, a product of the IM process, which cross-referenced targeted behavioral outcomes, performance objectives, behavioral mediators, and learning objectives (Bartholomew et al., 2006). Targeted behavioral outcomes for the original IYG curriculum included 1) abstinence from sex, and 2) maintenance of healthy relationships. These outcomes were also appropriate for all students, those who had not initiated sex, and also those who were sexually experienced but who could choose abstinence and healthy friendships in the future. Additionally, for sexually active youth, targeted behavioral outcomes included 3) correct use of condoms, 4) birth control, and 5) testing for HIV, STI, and pregnancy (Table 3). The original IYG curriculum was designed to impact psychosocial mediators related to these five behaviors which included knowledge, skills, self-efficacy, outcome expectations, perceived norms, normative beliefs, and social support. The IM matrices were reviewed by the *IYG*-Tech research team to determine the relevance and validity of the targeted behavioral outcomes and component behaviors (performance objectives), and behavioral mediators to current sexual health behaviors and determine whether modification or amendment would be required for the *IYG*-Tech version. The original matrices were found to be relevant to the new *IYG*-Tech program.

The learning objectives from the original IYG program (n = 193) were also reviewed by the research team to determine the extent to which each learning objective was covered in the original IYG lessons (whether full, partial, or limited/none) and in which types of lesson activities the learning objectives were covered (i.e. classroom and/or computer-based lessons). This analysis provided information on what content needed to be adapted to supplement the existing 8 IYG computer-based lessons. Of the 193 learning objectives listed in the original IYG planning matrices, 158 (88.9%) were covered in the 8 existing IYG computer lessons, either solely in the computer lessons (n = 82; 42.5%), more comprehensively in the computer lessons than the classroom (n = 23; 11.9%), or of equal comprehensiveness in the computer as in the classroom (n = 53; 27.5%) (Table 4). The classroom lessons provided the only content for 9 objectives (4.7%) and more comprehensive content for 26 objectives (13.5%) than provided on the computer. Learning objectives that were listed in the original *IYG* curriculum but required further focus in computer-based activity included skills training in refusing to have sex and avoiding unhealthy relationships (behavioral objectives 1 and 2), values clarification (e.g., setting one’s own personal limit with regard to sex, behavioral objectives 1 and 2), negotiating risk reduction strategies (e.g. negotiating condom use; behavioral objective 3), and health service utilization (e.g. getting STI testing if sexually active, behavioral objective 5).

Specifications for *IYG*-Tech lesson activities were listed in one of four categories: 1) as computer-based activities from the original IYG curriculum that were already adapted for the Internet and needed no change (n = 95), 2) computer activities from the original IYG curriculum that were already adapted for the Internet but required some minimal modification (n = 73), 3) computer activities adapted from pre-existing classroom activities in the original IYG curriculum that would be new Internet-based activities (n = 13), and 4) newly created Internet-based activities (n = 158).

Theoretical methods (e.g. modeling and guided practice to address skills and self-efficacy) and practical strategies (e.g. interactive and non-interactive animations, role model video, serials, video-based and photo-based interactive activities) informed activity content. Dynamic between-activity transitions and fact sheets were developed where more expansive content focus was required (e.g., skills practice for using condoms). Other design document specifications included lesson number and format, flow charts, storyboards, and interface design. The *IYG*-Tech lessons included branched logic to tailor content based on student gender, sexual experience, and sexual intentions. For students who are not sexually experienced or have few intentions to engage in sexual activity peer videos were framed to include messages reinforcing continued abstinence. For students who are sexually experienced, peer videos were framed to reinforce initiation of abstinence, risk reduction strategies, and STI testing.

Development of the design documents was an iterative process with components being presented to the teen advisory board for review and feedback on the appropriateness of activity content and language for credibility, authenticity, and appropriateness. For example, youth feedback assisted in the development of more realistic refusal skills simulations that portrayed more persistent peer pressure than originally scripted.

#### Step 4. Produce the new program

*IYG*-Tech comprises 13 lessons designed to be accessible to 8th grade students. The curriculum length was guided by the need to provide adequate content, suitability of implementation in a typical school semester, and prior empirical data suggesting minimal optimal dose for behavior change to be 13 lessons (Markham et al., 2012). Lessons are developed in HTML5, are approximately 35 minutes in length, and include interactive 2D exercises, quizzes, animations, peer video, and fact sheets that target mediators of sexual risk-taking. Selected lesson activities are tailored on gender, self-reported sexual experience and intentions. *IYG*-Tech is housed on a secure server in the University of Texas School of Public Health, public access pending results of efficacy field testing.

**Figure 3:**
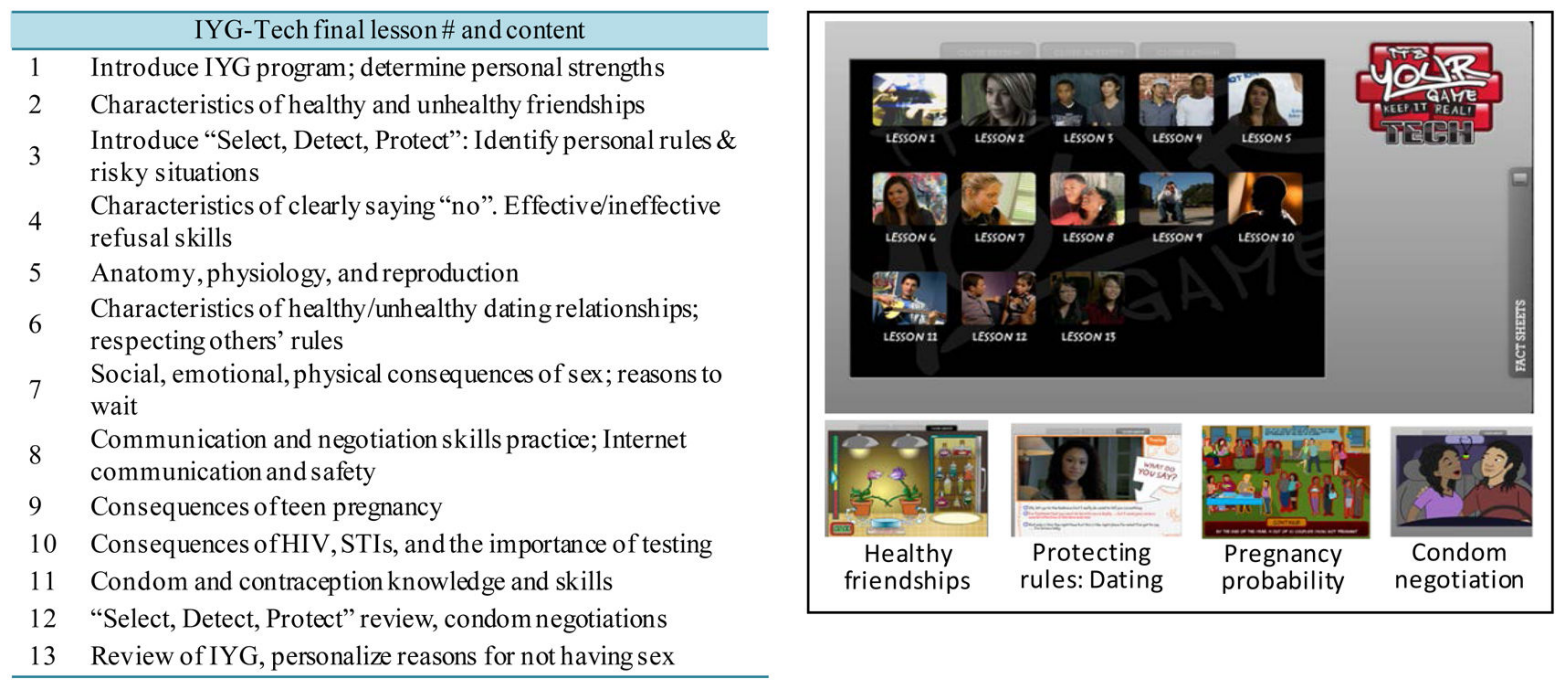
*IYG*-Tech final lesson content (tabled) and *IYG*-Tech screen captures illustrating lesson menu and selected new activities.

*IYG*-Tech lessons progress through a logical content sequence including characteristics of healthy friendships, setting and protecting personal limits, puberty and reproduction, characteristics of healthy dating relationships, consequences of sex (HIV, STI, and pregnancy), refusal skills training, the importance of testing if a person is sexually active, and skills training in condom and contraceptive use (Figure 3 and Table 5). The curriculum can be monitored by teachers who use a content management system (CMS) to list students and monitor progress and quiz scores and pre-post data. No sensitive information (entered by students in temporary journals or in interactive tailored activities) is available to the teacher. In-house alpha testing of the new Internet-based activities and full *IYG*-Tech program were conducted to ensure it conformed to the intentions of the designers and function appropriately prior to feasibility pilot testing. This included local and distance (Internet) access to the program.

### 2.4. PHASE 2: Feasibility Evaluation of *IYG*-Tech

A school-based pilot test of *IYG*-Tech was conducted to determine the feasibility of delivery in a typical school setting and its impact to inform readiness for further efficacy testing.

#### 

##### Study Design and Participants

A single group, pre-test post-test usability study of the *IYG*-Tech computer lessons was conducted at a Southeast Texas middle school with a convenience sample comprised of 7th and 8th grade students (n = 22), 13 – 15 years of age (14.4 ± .46 years), female (50%), and minority (91% Hispanic and 9% African American). Participation was voluntary; written parental permission and child assent were obtained. The study was approved by the University of Texas Institutional Review Board (HSC-SPH-09-0413).

##### Study Protocol

Students were recruited and participated in the usability testing during P.E or an elective class period at their school. Students tested the 13 *IYG*-Tech computer lessons over approximately a two week period (testing two lessons during a 90 minute class period each day). The students accessed the program in a classroom setting using laptop computers. Prior to accessing the program activities, students completed a demographic survey. Each student was then provided with a laptop computer containing the *IYG*-Tech lessons and head-phones and asked to complete each of the 35-minute Internet-based lessons individually. At the end of each lesson, each student completed a usability survey regarding that lesson. Lessons were observed by study personnel who logged problems (technical or content related) and provided assistance as required. Students received $50 for their participation. Students completed a demographic form, a usability survey for each lesson, and pre/post slider bar questions within the lessons.

##### Measurement and analysis

Usability parameters were as previously described for the lab-based usability study. Motivational appeal was also assessed on whether youth compared *IYG*-Tech favorably against their favorite computer game, other school lessons, and other computer-based school lessons, and other health lessons (less fun, as much fun, more fun). Perceived impact was assessed by asking youth if *IYG*-Tech would help them make healthy decisions on 29 parameters specific to the content of *IYG*-Tech including friendships, selecting and protecting personal rules, enabling better risk reduction communication and negotiation (clear no’s and alternative actions), increasing understanding of reproduction and the developing body, and lowering risk of pregnancy and STD’s using a 3-point Likert scale (“yes”, “no”, “don’t know”).

Psychosocial impact variables included perceived importance and self-efficacy in 6 sexual health domains including healthy dating relationships, protecting personal rules about sex, negotiating with others to protect your personal rules, consequence of pregnancy, consequence of getting HIV or STDs, protecting personal rules about sex. Importance and self-efficacy items were embedded in the *IYG*-Tech program as text questions accompanied by audio and provided immediately before and after each lesson. The response set was a 10-point Likert scale slider bar adapted from previously reported motivational enhancement methods (Velasquez et al., 2001). Youth were asked to rate how important each issue was (e.g. how important to you is PROTECTING your personal rules about sex, how important to you is avoiding getting HIV or sexually transmitted diseases?) on the scale ranging from “0” (not important at all) to 10 (extremely important). Youth were asked to rate their self-efficacy (e.g. How sure are you that you can PROTECT your personal rules about sex? How sure are you that you can negotiate with others to protect your personal rules?) on the scale ranging from “0” (not at all sure) to 10 (very sure). Data analysis is as previously described for usability testing.

##### Feasibility testing results

###### Usability

Usability ratings across all 13 *IYG*-Tech lessons indicated high agreement on likability for the entire program (91.8% – 100%) and for all individual activities (62.5% – 100%) (Table 6). Program components (graphic, audio, and transition features) were similarly highly rated (73.0% – 100%). Youth rated the program as easy to use (68.4% – 100%), of credible and correct content (80% – 100%), of acceptable duration (81.3% – 100%), and that the words were understandable (89.4% – 100%). However, youth also reported requiring assistance in using the program (up to 43% for a given lesson). *IYG*-Tech rated highly compared to other school lessons, health lessons, and school computer lessons (76.2% – 100%) but less so when compared to favorite computer games (36.9% – 81.2%). Youth indicated they would recommend *IYG*-Tech lessons to their class (72.2% – 94.7%) but provided lower agreement on playing lessons again (31.3% – 70%).

###### Perceived impact

Youth rated the lessons as being helpful in preparing them in a range of sexual health behaviors to reduce the risk of pregnancy and STD’s including making healthy choices, selecting personal rules, detecting challenges, protecting personal rules through negotiation, using clear “no’s” and alternative actions of avoidance and refusal (89.5% – 100%) (Table 7). Psychosocial determinants: As a result of lesson exposure students increased their perceived importance of, and confidence in, negotiating with others to protect their personal rules (*p* < .05) and significantly increased knowledge about what constitutes a healthy relationship (*p* < .05) (Table 8).

## 3. Discussion

After a program has been shown to be effective, a logical next step is its dissemination. Failure in dissemination, however, limits effective programs from reaching their full public health potential. Until recently, minimal progress had been made in the widespread dissemination of effective HIV, STI, and pregnancy prevention programs to youth, especially those targeted to middle schoolers (Kirby, 2007; Morrison, 2007; Lesesne et al., 2008; Rolleri, et al., 2008; Ingram et al., 2008; Noar et al., 2009). More recent initiatives are changing this landscape. These include the Office of Adolescent Health demonstration projects for the dissemination of evidence-based sexual health curricula (USDHHS, 2010) and the Administration for Children and Families (ACF) Family and Youth Services Bureau (FYSB) Care Act Personal Responsibility Education Program (PREP) funded trials (USDHHS, 2014). These initiatives are exploring the factors that facilitate successful implementation of evidence-based sexual health curriculum and the barriers that may prevent it.

*IYG*-Tech is an innovative contribution to sexual health education in that it is a completely online interactive curriculum, adapted from the effective *IYG* program. As an online curriculum, it is designed to mitigate conventional dissemination challenges of classroom-based curricula that can occur at the program, individual, and organizational level. While an Internet-based solution does not eradicate program level barriers, it does greatly optimize a facilitator’s ability to locate and acquire the program. Implementation of computer-based lessons can also vastly simplify facilitation, provided that school computer specifications meet broadband and connectivity requirements for the program.

Continued and repeated trainings and technical assistance have been the conventional method to overcome cited individual level barriers to classroom-based program implementation. These methods are designed to raise awareness, and reduce confusion about education policies (Levesque, 2000; Peskin 2011), increase skills and self-efficacy for teaching and adapting programs with fidelity, and reduce negative attitudes towards “science-based” approaches (Rolleri et al., 2008; Buston, 2002). To date, however, training has had limited effects on HIV program dissemination with adult populations (Rebchook et al., 2006; Harshbarger et al., 2006), requiring ongoing funding (Rolleri, et al., 2008; Philliber et al., 2008) and teacher willingness to attend trainings outside the school day (Basen-Engquist et al., 1994; Brink et al., 1991). A recent implementation trial of IYG in South Carolina, for example, reported 98.4% curriculum adherence and high quality implementation of the curriculum. However, this required an intensive technical assistance and training protocol with adequate funds to support these activities (Kershner et al., 2014). *IYG*-Tech is designed to provide high fidelity through a stand-alone curriculum requiring minimal-to-no facilitation. This delivery method may eliminate the barriers of teacher training and program fidelity. An Internet-based curriculum can provide confidential lesson content to students that are tailored on their sexual experience and intentions while reducing discomfort for facilitators who are not required to directly deliver what may be perceived as sensitive material.

An Internet-based delivery appears intuitively appealing as a channel to lower overhead and reduce costs (for purchasing, training, and implementing programs) to consumer school districts (Kirby, 2007; Landry et al., 2003; Noar et al., 2009; Rolleri et al., 2008) while also being a salient channel for “millennial” learners and timely channel given trends toward the adoption of technology by school districts in pursuit of great cost-effectiveness (National Center for Education Statistics, 2008). The upsurge of Internet connectivity in U.S. schools provides a natural substrate for the emergence of an eHealth curriculum like *IYG*-Tech. The adaptation of existing evidence-based programs (guided by well-established adaption frameworks) for access through interactive communication technology platforms (Card et al., 2011b; Wingood & DiClemente, 2008) provides precedent and guidance for developers to pursue this dissemination avenue without having to develop totally new programs.

The adaptation of IYG to *IYG*-Tech had a number of advantages including the strength of evidence of the original blended curriculum, the availability of the IYG creators in the adaptation process, and the existence of 8 original computer-based lessons and associated assets. Despite this, there were challenges in the creation of a digital version of the IYG curriculum that included ensuring that original learning objectives were fully represented, that a new Internet-based functionality of the original computer-based lessons could be achieved, and that the newly developed “proof-of-concept” lessons and prototype curriculum were acceptable to end users, feasible for delivery in the middle school setting, and impactful of mediators of sexual behavior.

The significant impact demonstrated in the evaluation of the Internet-adapted lessons included increased student perceptions of their friends having more positive beliefs about waiting to have sex, greater reasons for not having sex, greater self-efficacy for using condoms, and greater intentions to abstain from sex until marriage. These findings were also among the findings reported in the previous IYG field trials (Tortolero, 2010; Markham, 2012). Assessment of *IYG*-Tech impact was necessarily restricted to assessment of immediate pre-post change in psychosocial variables, but these results on psychosocial impact are encouraging, particularly considering the concatenated exposure to *IYG*-Tech necessitated in this study.

Advantages of the *IYG*-Tech learning experience include a one-to-one student-instructor (computer) ratio and a triangulation of strategies to target behavioral determinants including numerous examples of modeling of skilled behavior (e.g., saying no to having unprotected sex) and situational behaviors (e.g., making a choice to avoid high-risk situations and seeing the results of the choice) that influence normative perceptions as well as skills; confidential and personalized management of sensitive and potentially embarrassing issues; and individualized (tailored) intervention messages to specific characteristics of the users such as gender and sexual experience. Research studies have reported the potential of technology to impact sexual health knowledge (regarding decision-making, assertiveness, and communication), attitudes (regarding communication), self-efficacy, intentions, and self-reported behavior (Kann, 1987; Thomas et al., 1997; Lightfoot et al., 2007; Roberto et al., 2007; Kiene et al., 2006; Di et al., 2004). In a 3-arm evaluation (computer-based, small group, and control) of programs for delinquent youths in the juvenile justice system, Lightfoot and colleagues (2007) reported that the computer-based intervention was significantly more effective, at 3-month follow-up, in lowering frequency of sexual intercourse and that both conditions (computer-based and small group) significantly reduced the number of sex partners than the control condition. In an evaluation of 6 computer-based activities in 9 rural high schools, Roberto and colleagues (2008) found significant effects on knowledge about pregnancy and STIs, condom self-efficacy, attitudes toward waiting to have sex, and perceived susceptibility to HIV compared to controls. Such findings have prompted calls for the continued development, testing, and ultimate dissemination of digital (computer-based and social media) interventions to push the research agenda forward (Noar, 2009; Allison et al., 2012).

Positive ratings across usability parameters and perceived benefits of *IYG*-Tech to sexual health were commensurate with those of usability testing of the original *IYG* computer-based lessons (Shegog et al., 2007). The positive effect on attitudes to using computers in the classroom and the positive acceptability of *IYG*-Tech are likely related, at least in part, to the purposeful use of game strategies that have been successful in enhancing knowledge, self-efficacy, attitudes, and behaviors in varied education and health domains. The *IYG* computer lessons, purposefully designed to be immersive and motivational, include multiple gaming and edutainment elements in support of current initiatives for the development and formal research of socially conscious games for health (Health Games Research, 2013), and extend a growing body of evidence indicating the efficacy of computer-based applications in impacting health behavior change in youth (Papastergiou, 2009; Primack et al., 2012; Hieftje et al., 2013) and, more specifically, the early research on games for pregnancy prevention (“The Baby Game!” and “Romance!”) shown to enhance motivational appeal, knowledge, and intentions (Paperny & Starn, 1989).

While encouraging, the results reported here need to be interpreted in the context of study limitations that include artificially abbreviated exposure to the 8 Internet-adapted *IYG* computer-based lessons (lab-based “proof-of-concept” usability test) or the full *IYG*-Tech curriculum (school-based feasibility test) with immediate post-test; the single-group pre-test/post-test study design without a control group to mitigate threats to internal validity; the purposive sampling of participants; and the potential of demand characteristics to influence youth responses and feedback. Pilot feasibility studies of the type reported here are necessarily limited and designed to provide a measure of confidence that broader field trials are warranted and to highlight “red flags” (ranging from program bugs to deleterious usability or psychosocial impact) that suggest field testing is premature. Further, while most schools have internet connectivity, they vary in the number of available computers, Internet reliability, and bandwidth. The study described here cannot adequately address these challenges which may become apparent in broader efficacy trials. Of course, the digital approach to sexual health may provide an insufficient solution to organizational barriers. These barriers include the concern of school officials about negative community reactions and perceived controversy of sex education (Landry et al., 2000), a perceived lack of support from outside stakeholders (e.g. parents) for teaching this topic (Rolleri, et al., 2008; Paulussen et al., 1994; Buston et al., 2002) and pressure to devote class time to academic priorities rather than health topics. It is important to note that a successful implementation (whether it is Internet or conventional classroom lessons, or a blended approach) needs to address such organizational barriers to be successful. *IYG*-Tech is being evaluated in a randomized controlled trial in a large urban school district. Research results will be important to demonstrate the relative efficacy of this innovative technology-based curriculum compared with usual care, as well as to determine the cost-effectiveness implications of this implementation and dissemination strategy.

## 4. Conclusion

The development and testing of *IYG*-Tech reported here have demonstrated the usability and appeal of this curriculum among middle school students, suggesting that it may be a feasible delivery channel. Features of this approach include adherence to a rigorous, stepped adaptation protocol with adaptation steps informed by iterative prototype testing (usability, feasibility, impact), providing empirical evidence to inform each subsequent development step. This work provides a guide for future work on adaptation of evidence-based programs onto a communication technology platform.

## Figures and Tables

**Table 1:** Participant usability ratings for the “proof-of-concept” lessons adapted for the Internet from the original 8 IYG computer lessons.

Construct	Item	Feasibility results (range of rounded % agreement)
Likability (Like a little or a lot)	Entire program	94
	Activities (n = 79 across all lessons)	n/a
	Pictures/colors	94
	Sounds	88
	Buttons	94
	Cartoons	97
	Videos	97
	Transitions	94

Ease of use (kind of easy or very easy)	Entire program	88
	Directions	100

Credibility	Information is right (correct)	91
	Information is trustworthy	97

Acceptability	Duration/Time-on-task was “just right”	66

Understandability	Understands words	88
	Needs help	24

Motivational Appeal	Would recommend to my class	79
	Would play again	45

Perceived Impact	Helps make healthy choices	91

**Table 2:** Change in psychosocial determinants of sexual behavior and attitudes toward computers for learning following exposure to internet-based *IYG* computer lessons.

Psychosocial variable	*n*	Pre-test *M* (SD)	Post-test *M* (SD)	Change *M* (SD)	*t*
Beliefs about abstinence until marriage	28	3.01 (.65)	2.94 (.78)	−.07 (.57)	−.664
Beliefs about waiting to have sex	31	3.20 (.61)	3.22 (.61)	.02 (.55)	.163
Perceived friends’ beliefs about waiting to have sex	31	2.45 (.69)	2.74 (.52)	**.29 (.68)**	2.390*
Perceived friends’ sexual behaviors	29	1.64 (.71)	1.66 (.78)	.03 (.64)	.219
Reasons for not having sex	31	4.00 (2.38)	5.19 (2.88)	**1.19 (1.85)**	3.589**
Reasons for having sex	30	.73 (.83)	.73 (1.43)	.00 (1.68)	.000
Self-efficacy to refuse having sex	26	3.19 (.65)	3.24 (.73)	.05 (.69)	.407
Condom knowledge	31	.68 (.35)	.75 (.33)	.08 (.36)	1.157
Condom attitudes	30	3.28 (.68)	3.14 (.98)	−.13 (.72)	−1.015
Perceived friends’ beliefs about condoms	29	2.87 (.81)	3.03 (.92)	.16 (.89)	.097
Self-efficacy to use condoms	24	1.31 (.56)	1.52 (.47)	**.21 (.44)**	2.345*
Perceived likelihood of having oral sex in the next year	31	1.90 (1.14)	1.71 (1.24)	−.19 (.83)	−1.293
Perceived likelihood of having vaginal sex in the next year	31	1.84 (1.13)	1.81 (1.33)	−.03 (.91)	−.197
Perceived likelihood of abstaining until the end of high school	29	3.12 (1.52)	3.48 (1.72)	.28 (1.56)	.955
Perceived likelihood of abstaining until marriage	29	2.83 (1.61)	3.59 (1.34)	**.76 (1.22)**	3.363**

**Attitude toward computers for learning**					
While studying computers, the time passes quickly.	23	2.76 (.59)	2.275 (.88)	**−.39 (.84)**	−2.237*
I learn quickly while studying with computers.	23	2.80 (.77)	2.448 (.78)	**−.43 (.95)**	−2.206*
I feel comfortable while studying with computers.	23	2.76 (.52)	2.586 (.68)	−.17 (.78)	−1.073
Learning with computers increases my success.	23	2.44 (.71)	2.310 (.81)	−.13 (.87)	−.720
Learning from computers increases my confidence.	23	2.00 (.76)	2.344 (.72)	**.35 (.57)**	2.912**
Computers make me eager to study more.	23	2.08 (.91)	2.366 (.81)	.13 (.76)	.826
At first, learning with computers seems enjoyable but later I am bored.	23	2.12 (.78)	2.266 (.78)	.04 (.71)	.295
Instruction with computers is very enjoyable.	23	1.92 (.86)	2.413 (.78)	**.48 (1.08)**	2.121*
I would like to learn all my school courses with computers.	23	2.28 (.76)	2.233 (.89)	−.04 (.93)	−.225
I learn easily with colorful graphics and animation.	23	2.44 (.82)	2.466 (.78)	−.04 (.93)	−.225

Note:

a All scales positively coded, except likelihood to have oral and vaginal sex.

* *p* < .05,

** *p* < .01.

**Table 3:** Targeted behavioral determinants of *IYG*-Tech.

**All students** Students will not have sex. Students Will Have Healthy Relationships With Their Friends, Girlfriends, or Boyfriends. **For students who ARE sexually active OR considering having sex.** Students will use condoms correctly and consistently when having sex. Students will use birth control correctly and consistently when having sex. Students will get tested and counseled for HIV, STDs, and Pregnancy.

**Table 4:** Curriculum content analysis. Learning obectives covered by each lesson type.

#	Category	Definition	Objectives n (%)
1	Classroom = computer	Equally in classroom and computer lessons	53 (27.5)
2	Classroom only	In classroom but NOT in the computer	9 ( 4.7)
3	Computer only	In the computer but NOT in the classroom	82 (42.5)
4	Classroom > computer	More comprehensively in the classroom than in the computer	26 (13.5)
5	Computer > classroom	More comprehensively in the computer than in the classroom	23 (11.9)
	Total		193 (100.0)

**Table 5:** *IYG* -Tech final lesson learning objectives by outcome behaviors and mediators.

Mediator	Learning objectives covered in *IYG*-Tech
**1. Students will not have sex**	
Knowledge of…	Reproductive system (functional anatomy)/Types of sex (oral, anal, vaginal)/Consequences of sex (physical, emotional, social)/“Personal limits or rules”/Situations (places, peers, times) & signs (loss of “control”, pressure, feelings) that may make it hard to say no to sex;/Characteristics of clear refusal skills/Abstinence as being the only 100% effective way of avoiding HIV, STD, or getting pregnant./Pressures/influences (social, peer, partner, media) to have and not have sex/Communicating your rules to friends &/or partner & reasons why this is important/Alternate activities (e.g. movies, pizza, meet friends) and ways to suggest these/Avoidance strategies/Signs as cues to use refusal strategies/Personal rules regarding sex & intimate behaviors.
Skills and self-efficacy to…	Decide to not have sex/Identify signs & situations that may make it hard to say no to sex (e.g., peer pressure, social situations, when you really like the person) & use refusal skills/Communicate your personal rules to friends &/or partner/Identify & listen to signs and situations that may make it hard to say no to sex & avoid those situations (e.g. physically avoid the situation; use refusal/negotiation skills)/Suggest an appropriate alternative activity to sex.
Outcome expectations that…	The decision to not have sex will reduce the risk of getting HIV, STDs or becoming pregnant/Communicating your personal intentions and limits will lead to a better relationship with your partner/Communicating personal rules & intentions will decrease risk of HIV/STD & pregnancy/A healthy relationship is not predicated on sexual activity/Avoiding a high risk situation, alternative activities, &/or use of appropriate refusal skills will lead to successful abstinence without jeopardizing interpersonal relationships & reduce the risk of getting HIV, STDs or becoming pregnant./Deciding to not have sex will lead to increased self-respect/Use of refusal skills will keep you from doing things you don’t want to do (non-sex related) without jeopardizing friendships.
Perceived norms that…	Teens communicate their personal limits to friends/Teens communicate their personal limits regarding sex to their partners/Most middle school students do not have sex/Most middle school students feel it is important to not have sex.
Normative beliefs that…	Significant others approve and respect your refusing to have sex/People may have different personal rules regarding different behaviors/Friends approve of you communicating your personal rules/Your partner approves of you communicating your limits/Others approve of you recommending alternate activities to sex/Significant others approve and respect you refusing to do things that you choose not to do (non-sex related behaviors)/Significant others approve and respect your decision to avoid situations that may make it hard to say no to sex/Most parents feel it is important to practice refusal strategies to not have sex.
Social support to…	Use alternate activities/Decide to not have sex/Establish and communicate your personal intentions and limits/Avoid and/or help identify signs of risky situations/Use refusal strategies to not have sex.
**2. Students Will Have Healthy Relationships With Their Friends, Girlfriends, or Boyfriends**	
Knowledge of…	Characteristics of healthy & unhealthy relationships/Healthy & unhealthy ways you behave in a relationship/Pressures & influences (social, peer, media) to have and not have healthy relationships/Expectations about healthy relationships/Ways to communicate expectations about relationships to friends & partner/Relationships with friends & partners that are not healthy/Situations where you come in contact with friends or partners that are not healthy/Strategies to avoid friends or partners that are not healthy/Alternative activities to being in unhealthy relationships.
Skills and self-efficacy to…	Evaluate relationships/Recognizing signs of unhealthy relationships/Have healthy relationships/Listen to your friends’ or partner’s expectations in relationships/Avoid unhealthy relationships/Communicate expectations in relationships/Engage in alternative activities to being in unhealthy relationships.
Perceived norms that…	Most peers can have healthy relationships.
Social support to…	Set and communicate expectations for healthy relationships./Evaluate relationships/Decide to have a healthy relationship/Avoid unhealthy relationships.

For students who ARE sexually active OR considering having sex.	
Mediator	Learning objectives covered in *IYG*-Tech
**3: Students will use condoms correctly and consistently when having sex.**	
Knowledge of…	Reproductive system (functional anatomy)/HIV/STDs (Transmission & symptoms)/Consequences of not using a condom if you have sex/Condoms in reducing the risk of HIV, STDs, & pregnancy/Types of condoms and relative pros & cons/Condom evaluation, application, & safe removal/Pressures & influences (peer, media, social) for condom use & none use/Places to obtain or buy condoms/Reasons for, and importance of, communication of intentions to use a condom to a partner/Protected and unprotected sex/Legality of condom purchase/Ways to purchase, carry, & protect a condom/Communication about intentions & negotiation to use condoms/Personal rules about condom use/Reasons that condoms are important with regular as well as casual partners.
Skills and self-efficacy to…	Evaluate, apply, and safely remove a condom/Decide to use condoms/Buy or obtain a free condom/Carry a condom/Negotiating condom use: Communicate intentions to use condoms, hear partner’s intentions, negotiate condom use, agree on condom use or to not have sex/Apply a condom correctly/Use a condom with regular partners as well as with casual partners.
Outcome expectations that…	Deciding to use condoms & using them correctly reduces the risk of getting HIV, STIs, pregnancy/A-priori agreements about using condoms (or not having sex) will increase the chances that these occur/Condom negotiation increases condom use with sex/Communicating intentions to use condoms with sex increases their use/Using condoms every time you have sex (even with regular partners) reduces the risk of getting HIV, STD’s, & pregnancy/Carrying a condom increases the chances of using one when having sex.
Perceived norms that…	Most sexually active teens buy or obtain free condoms, carry condoms, feel it is important to carry condoms, communicate their intentions to use condoms with their partners, & choose to not have sex if a condom is not used/Most sexually active teens who decide to use condoms are more likely to use them & to successfully negotiate their use/Partners should be willing to use condoms/Teens use condoms with regular & casual partners and feel this is important.
Perceived barriers to…	Recognizing that the benefits of using condoms outweigh the risks and side effects of not using condoms/Buying or obtaining a free condom (e.g., money, transportation), carrying a condom, and using condoms consistently over time.
Social support to…	Buy or obtain a free condom/Support your decision to use condoms/Support your decision to carry a condom, negotiate the use of a condom, making an agreement to use condoms or not have sex, & use condoms consistently over time.
**4: Students will use birth control correctly and consistently when having sex.**	
Knowledge of…	Birth control options & methods (including advantages and disadvantages)/Guidelines for effective and consistent use of chosen birth control (plan ahead, take every day)/Both partners being responsible for use of non-hormonal birth control when sexually active.
Outcome expectations that…	Making an informed choice about birth control will decrease risk of pregnancy/Effective use of birth control along with condoms decreases risk of pregnancy.

For students who ARE sexually active.	
Mediator	Learning objectives covered in *IYG*-Tech
**5: Students will get tested and counseled for HIV, STDs, and Pregnancy**	
Knowledge of…	Types of STDs & modes of transmission/Types of tests available (different procedures; home/clinic)/Time taken to get test results./Limitations of tests (e.g., timing premature to detect HIV, pregnancy; false negative/positive; no counseling with home tests)./Places to get testing & counseling (incl. business hours, phone numbers)/Healthcare needs addressed & appropriate provider/Making an appointment (time & date)/Obtaining results (e.g. phone, in person)/Way to contact partners you have had (e.g. phone #, address).
Skills and self-efficacy to…	Decide to get tested and rationale for decision./Make and keep an appointment/Obtain test results/Cope with results/Engage in conversation w/your healthcare provider & get healthcare/Notify partner(s)/Plan ahead (use reminders)/Maintain testing behavior.
Perceived susceptibility that…	Any sexually active teen can get HIV/STD or become pregnant/impregnate partner/Current absence of symptoms does not ensure that you are not infected or pregnant/If you continue to be sexually active, you need to maintain testing behavior/Teens have a certain prevalence of HIV/STD & pregnancy.
Perceived severity of…	Consequences of teen pregnancy, STDs, & HIV/Consequences of particular STDs if you don’t obtain healthcare follow up.
Perceived norms that…	It is normal, expected, and responsible for teens who have sex to get tested for HIV/STD and pregnancy/People who are sexually active maintain testing behavior over time./Individuals who have HIV/STD or who are pregnant obtain healthcare/It is an individual’s responsibility to make past & future partners aware of their status.
Perceived benefits of…	The importance of knowing your status (& overcoming barriers such as denial or not wanting to know)/That getting tested outweighs barriers/That knowing your status is beneficial for health & peace of mind of self & partner, can result in early treatment (if positive results), & helps to reduce progression of symptoms (HIV/STD) or to have a healthier baby (if pregnant)/That taking responsibility for health of self & partner by getting tested over time is helpful, worthwhile, useful, the mature thing to do./Feeling mature and responsible for keeping the appointment, getting the test results, and notifying partner(s).
Perceived barriers to…	Getting tested that include cost, access, confidentiality, type of test, stigma, and embarrassment/Develop mitigating strategies/List free or low-cost testing sites (cost)/Recognize that testing is not a one-time occurrence/List your available times for testing/Be aware of your schedule (don’t double book)/Notify past and future partners/Identify ways to overcome barriers.
Social support to…	Your decision to get tested/Helping you make appointment, keep the appointment, and accompany you to get the results/Accompanying you to get treatment/Help you cope with treatment/Help and support you to notify partners/Help and support continued testing behavior.

**Table 6:** Participant usability ratings for 13-lesson *IYG*-Tech prototype Internet-based curriculum.

Construct	Item	Feasibility results (range of rounded % agreement)
Likability (Like a little or a lot)	Entire program	92 – 100
	Activities (n = 79 across all lessons)	63 – 100
	Pictures/colors	84 – 100
	Sounds	80 – 100
	Buttons	83 – 100
	Buildings and people	88 – 100
	Cartoons	81 – 100
	Videos	73 – 100
	Transitions	88 – 100

Ease of use (kind of easy or very easy)	Entire program	100
	Activities (n = 79 across all lessons)	69 – 100
	Directions	94 – 100

Credibility	Information is right (correct)	88 – 100
	Information is trustworthy	80 – 100

Acceptability	Duration/Time-on-task was “just right”	81 – 100

Understandability	Understands words	89 – 100
	Needs help	0 – 44

Motivational Appeal	Would recommend to my class	72 – 95
	Would play again	31 – 70
	As much or more fun as my favorite computer game	37 – 81
	As much or more fun as other school lessons	77 – 100
	As much or more fun as other school computer lessons	78 – 100
	As much or more fun as other health lessons	88 – 100

Perceived Impact	Helps make healthy choices	89 – 100

**Table 7:** IYG curriculum content by lesson and percent agreement on perceived impact.

	Lesson # and content	The information provided in this lesson will help me…	n (%)*	
			n	%
1	Introduction, determine personal strengths.	make healthy choices	15	93.8

2	Characteristics of healthy and unhealthy relationships.	know how to tell real friends	10	100.0
		be a real friend	10	100.0
3	Introduce “Select, Detect, Protect”: Identify personal rules & risky situations.	select personal rules regarding healthy friendship	17	89.5
		detect challenges to my personal rules	18	94.7
		protect my personal rules	18	94.7

4	Characteristics of clearly saying “no”. Effective/ineffective refusal skills.	use clear “no’s”	18	100.0
		use alternate actions	10	100.0
		avoid/refuse	17	100.0

5	Anatomy, physiology, and reproduction.	know how my body develops	19	95.0
		know how reproduction works	19	95.0
		know what sex is	20	100.0
		know the consequences of sex	19	95.0

6	Characteristics of healthy and unhealthy dating relationships; respecting others rules.	have healthy relations	18	100.0
		play by my “rules”	18	100.0

7	Social, emotional, physical consequences of sex; reasons to wait.	protect my “rules”	14	100.0

8	Communication & negotiation skills practice; Internet communication & safety.	to negotiate to protect my rules	19	100.0

9	Consequences of teen pregnancy.	reduce pregnancy risk	18	100.0
		make responsible decisions	18	100.0

10	Consequences of STI/HIV & the importance of testing.	make responsible decisions	21	100.0
		reduce STD risks	20	95.2

11	Condom and contraception knowledge and skills.	make responsible decisions	18	100.0
		reduce pregnancy & STD risk	18	100.0

12	“Select, Detect, Protect” review; condom negotiations.	select my personal rules	18	100.0
		detect challenges to my personal rules	18	100.0
		protect my personal rules	18	100.0
		to negotiate to protect my rules	16	94.1

13	Review of IYG; personalize reasons for not having sex.	make safe choices	20	100.0
		remember	20	100.0

Note:

* Remainder responses were “don’t know”.

**Table 8:** Immediate within-lesson psychosocial impact (importance of topic and self-efficacy) by content domain^1^–^3^.

Psychosocial variables	n	Pre-test Mean (SD)	Post-test Mean (SD)	Change Mean (SD)	t	*p*	Delta Signed Rank	Sign Rank *p*
**Healthy Dating Relationships (Lesson 6)**								
How sure are you that you know what makes a healthy dating relationship?	19	7.79 (2.52)	9.81 (.77)	2.01 (2.31)	3.80	.0013	39	**.0005**
How important to you is understanding what makes a healthy dating relationship?	19	9.23 (1.34)	9.82 (.54)	.53 (1.23)	1.86	.0753	18.5	**.0234**
**Protecting personal rules about sex (Lesson 7)**								
How sure are you that you can PROTECT your personal rules about sex?	13	7.95 (3.56)	9.53 (.89)	1.58 (3.01)	1.90	.082	7.5	.1563
How important to you is PROTECTING your personal rules about sex?	13	8.75 (2.97)	9.31 (1.86)	.56 (1.79)	1.13	.2806	4.5	.4375
**Negotiating with others to protect your personal rules (Lesson 8)**								
How sure are you that you can negotiate with others to protect your personal rules?	16	8.34 (1.92)	9.94 (.25)	1.59 (1.82)	3.52	.0039**	22.5	**.0039**
How important to you is negotiating with others to protect your personal rules?	16	8.72 (1.98)	10.00 (.00)	1.28 (1.98)	2.58	.0210	14	**.0156**
**Consequence of pregnancy (Lesson 9)**								
How sure are you that you understand the consequences of pregnancy?	11	8.98 (3.00)	9.88 (.33)	.89 (3.06)	.97	.3558	2	.6250
How important to you is avoiding the consequences of pregnancy?	11	9.65 (1.15)	9.53 (1.50)	−.119 (1.96)	−.20	.8444	−.5	1.0000
**Consequence of getting HIV or STDs (Lesson 10)**								
How sure are you that you understand the consequences of getting HIV or sexually transmitted diseases?	9	8.78 (1.72)	10.00 (.00)	1.22 (1.72)	2.12	.0668	5	.1250
How important to you is avoiding getting HIV or sexually transmitted diseases?	9	8.62 (2.17)	10.00 (.00)	1.38 (2.16)	1.92	.0915	5	.1250
**Protecting personal rules about sex (Lesson 12)**								
How sure are you that you can PROTECT your personal rules about sex?	19	9.39 (1.77)	9.99 (.03)	.61 (1.77)	1.49	.1547	8.5	.0938
How important to you is PROTECTING your personal rules about sex?	19	9.79 (.44)	9.47 (1.57)	−.32 (1.50)	−.93	.3623	−1.5	.8125

Note:

1 All scales positively coded except likelihood to have oral and vaginal sex.

2 Sample is restricted to subjects with valid responses for both pre- and post-sliders for a given item.

3 Sample size was 1 or 2 for the following items: know what makes a good friend? (lesson 1), having good friendships to you? (lesson 2), that you have personals rule to live by? (lesson 3), that you can PROTECT your personal rules? (lesson 4), that you understand how reproduction works? (lesson 5), that you can reduce your risk of pregnancy or getting HIV and other sexually transmitted diseases? (lesson 11).
